# Combining multispectral and high-resolution 3D imaging for leaf vein segmentation and density measurement

**DOI:** 10.3389/fpls.2025.1560220

**Published:** 2025-03-10

**Authors:** Yi-Hong Liao, Song Zhang

**Affiliations:** School of Mechanical Engineering, Purdue University, West Lafayette, IN, United States

**Keywords:** leaf vein segmentation, vein density, multispectral imaging, 3D imaging, structured light, high throughput, precision agriculture

## Abstract

Accurate leaf vein segmentation and vein density (VLA) measurement are crucial for understanding plant physiology. Traditional 2D imaging techniques often require labor-intensive and destructive processes, such as leaf flattening or chemical clearing, thereby limiting their practicality for high-throughput applications. In this study, we present a novel framework that integrates multispectral and high-resolution 3D imaging to enhance leaf vein segmentation and VLA measurement. By leveraging digital fringe projection, our system captures grayscale, multispectral, and 3D topographical data within a unified coordinate system. The integration of 3D information improves vein detection, particularly in low-contrast regions, while also enabling direct and accurate measurements of leaf area, vein length, and VLA. However, this approach also introduces some false positives in vein segmentation due to mesophyll surface variability. Despite these challenges, our high-resolution 3D imaging method shows significant potential for non-invasive phenotyping and trait assessment in complex, unstructured environments.

## 1 Introduction

Leaf veins play a critical role in plant physiology, offering valuable insights into the structure and function of a plant’s vascular system. For example, sugar maple (*Acer saccharum*) is economically significant for producing maple syrup and high-quality lumber, as well as for its importance in the landscape and forestry industries. A substantial portion of the sugar maple leaf’s hydraulic resistance resides within its major veins, directly impacting photosynthesis (Sack et al., 2004). Greater vein length (VL) per unit leaf area (VLA, also known as vein density) has been linked to improved sugar maple seedling growth, emphasizing the importance of veins in supporting the species’ development through enhanced hydraulic efficiency (Zhu et al., 2020b). Given its considerable economic value, understanding the venation system of sugar maple is vital for optimizing growth and yield.

The health and productivity of sugar maple trees, like many other plants, are closely tied to the efficiency of their venation system, which serves several essential functions, such as transporting water (Brodribb et al., 2010), nutrients (Zhang et al., 2015), and sugars (Haritatos et al., 2000; Dinant and Le Hir, 2022), in addition to providing mechanical support (Rolland-Lagan and Prusinkiewicz, 2005; Peng et al., 2022) and enhancing resistance to leaf damage (Choong, 1996). Traits derived from leaf veins significantly influence leaf functionality. For example, VLA is closely associated with water transport efficiency (Brodribb and Feild, 2010; McKown et al., 2010; Sommerville et al., 2012), hydraulic resilience (Scoffoni et al., 2011; Kawai and Okada, 2018), and photosynthetic capacity (Brodribb et al., 2007; Schneider et al., 2017; Li et al., 2018; Sack and Scoffoni, 2013; Price et al., 2014). The strategic arrangement and density of veins optimize water transport and gas exchange, which are critical for maintaining leaf water balance and maximizing CO_2_ assimilation. Additionally, a higher VLA improves hydraulic resilience by providing alternative water flow pathways in case of vein damage, thus supporting consistent physiological function (Scoffoni et al., 2011).

To measure VLA, leaf vein segmentation is essential. Over the years, various methods for vein segmentation have been developed, ranging from 2D image-based techniques to 3D geometry-based approaches. 2D methods can be broadly classified into non-learning and learning-based approaches. Nonlearning methods include independent component analysis (ICA) (Li et al., 2006), grayscale morphology combined with Otsu thresholding (Zheng and Wang, 2010), Gabor filters (Katyal and Aviral, 2012), Hessian matrix for venation detection (Salima et al., 2015), and the object-oriented classification (Zhu et al., 2020a). For better flexibility and adaptability to dynamic environments and diverse datasets, learning-based approaches, particularly deep learning, have emerged as powerful alternatives, offering enhanced flexibility and automation. For instance, convolutional neural networks (CNNs) have been employed for leaf vein segmentation (Xu et al., 2021; Li et al., 2022; Iwamasa and Noshita, 2023; Cai et al., 2024).

Both non-learning and learning-based methods have demonstrated effectiveness in vein segmentation. However, they encounter challenges, particularly in distinguishing veins from mesophyll under poor lighting conditions or when vein and background colors are similar. As a result, these methods often depend on high-contrast images obtained through leaf flattening or chemical clearing, which are labor-intensive and time-consuming processes (Li et al., 2022). Leaf clearing alone can take several days (Bruzzese and Hasan, 1983) or even weeks to months (Richardson and Lichtman, 2015). These procedures, which involve handling and flattening, risk damaging leaves and compromising measurement accuracy (Vasco et al., 2014). Similarly, while X-ray imaging can be effective, it requires specialized facilities, involves lengthy measurement times, and may damage delicate structures (Iwamasa and Noshita, 2023), with its accuracy being influenced by leaf thickness and water content. Moreover, 2D image-based methods lack the topographical data needed for direct VLA measurements. Traditional methods involve flattening and scanning leaves using a flatbed scanner at a known dpi (Bühler et al., 2015), which are not suitable for high-throughput applications, as each leaf must be collected from the field, flattened, and scanned individually. Furthermore, these methods are prone to inaccuracies caused by leaf damage and incomplete flattening, particularly when dealing with highly curved leaves. These limitations underscore the need to explore 3D geometry-based approaches.

3D geometry-based approaches offer an alternative by utilizing spatial information to distinguish veins from the surrounding mesophyll. For instance, Sun et al. (2011) employed laser scanning for vein identification through curvature analysis, while Zhang et al. (2018) used photometric stereo to reconstruct 3D features. However, these methods are often time-consuming, sensitive to noise, and dependent on assumptions regarding leaf surface properties, which limits their scalability in high-throughput applications. Li et al. (2021) and Balasubramaniam et al. (2023) employed fringe projection to capture a 3D image of a spinach leaf, segmenting the veins by applying a threshold to the gradient of the depth image. However, this approach only achieved a rough segmentation of the largest vein. Similarly, Wen et al. (2024a, b) utilized 3D digitizers and scanners to obtain the point cloud of maize leaves. However, their method only identified vein points by selecting the middle vertices of each row in the leaf point cloud. Despite these challenges, they have shown promising results on relatively flat leaves with pronounced vein geometry variations.

Consequently, we hypothesize that integrating high-speed, high-resolution 3D imaging with 2D techniques could improve leaf vein segmentation, particularly under suboptimal imaging conditions, while also enabling accurate, direct VLA measurement. Unlike 2D methods that depend on idealized setups, 3D imaging can provide additional topographical information that may enhance vein detection efficiency and robustness. Therefore, we propose a leaf vein segmentation framework that integrates 2D grayscale and multispectral imaging with high-resolution 3D imaging. By leveraging differences in topographical properties between veins and mesophyll (Wang et al., 2020; Li et al., 2023), our framework aims to achieve accurate vein segmentation and VLA measurement without the need for leaf flattening.

To test this hypothesis, we developed a multispectral and 3D imaging system leveraging digital fringe projection to capture grayscale, multispectral, and high-resolution 3D images within a unified coordinate system. We employed existing 2D non-learning and learning-based techniques on grayscale and multispectral images, while also introducing a novel 3D vein segmentation approach. As anticipated, the results demonstrated enhanced segmentation performance, particularly in identifying veins within lowcontrast regions. However, this improvement also led to an increase in false positives due to unpredictable geometric variations in the mesophyll. Despite this limitation, the high-resolution 3D imaging facilitated direct trait measurements, allowing us to calculate leaf area (LA), vein length, and VLA directly from the 3D geometry. Validation against traditional 2D flatbed-scanned measurements confirmed the accuracy of our approach, underscoring the potential of 3D imaging for accurate VLA assessment in complex, unstructured environments.

## 2 Materials and methods

The design of a multispectral and 3D imaging system capable of capturing both multispectral images and high-resolution 3D point clouds of the measurement surface is introduced. The digital fringe projection (DFP) technique employed for high-resolution 3D reconstruction is then detailed. A method for estimating the spectral reflectance of leaves is subsequently presented. Next, a leaf vein segmentation framework that integrates 3D geometry, grayscale images, and multispectral data is described. Finally, a method for directly calculating VLA using 3D geometry is introduced.

### 2.1 Multispectral and 3D imaging system

**Figure 1:**
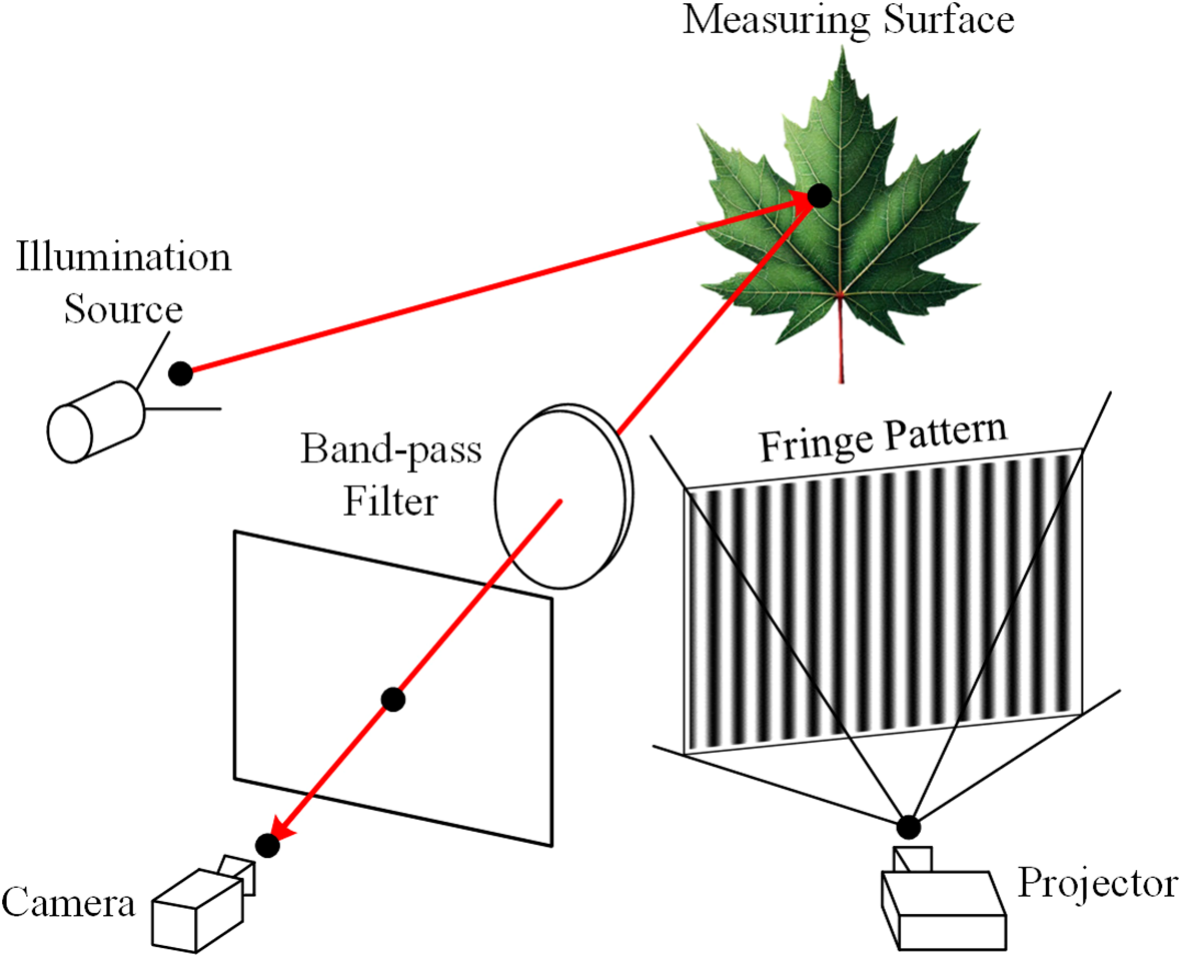
Schematic diagram of the multispectral and 3D imaging system.

To obtain both 2D and 3D information, a system that integrates multispectral and 3D imaging is designed, as illustrated in **Figure 1**. The system comprises a camera for capturing grayscale images, multispectral images, and fringe patterns for 3D reconstruction, along with a projector that both projects patterns for the 3D reconstruction process and provides illumination for grayscale imaging. The illumination source includes a point light source, a plano-convex lens, and a diffuser to generate wide spectral band diffuse illumination. A band-pass filter positioned in front of the camera enables it to capture images at different wavelengths by selectively filtering the incoming light.

For capturing multispectral images, various band-pass filters are sequentially placed in front of the camera while the projector is turned off. Conversely, when capturing grayscale images and fringe patterns for 3D reconstruction, the band-pass filters are removed, and the illumination source is switched off. The system’s design ensures that the multispectral images, grayscale images, and 3D point clouds are all captured within the same camera coordinate system, thereby eliminating the need for coordinate alignment. This is crucial, as even a small misalignment between the images could result in inaccuracies in leaf vein segmentation.

### 2.2 Digital fringe projection technique

The digital fringe projection (DFP) technique is a type of structured light 3D measurement method. As shown in **Figure 1**, a structured light system is formed by combining a camera and a projector. The projector casts fringe patterns onto the object, which are then distorted by the object’s shape and captured by the camera from a different viewpoint. This setup is analogous to a binocular system, where the projector functions as an inverted camera (Zhang and Huang, 2006). As a result, both the camera and the projector can be described using the linear pinhole lens model:


$$\gamma^{c}[u^{c},v^{c},1{]}^{T}=K^{c}\left[I,0\right][x,y,z,1{]}^{T},$$

(1) $$\gamma^{p}[u^{p},v^{p},1{]}^{T}=K^{p}M[x,y,z,1{]}^{T}.$$

Here, *γ* denotes a scaling factor, [*u,v*] represent undistorted pixel coordinates, ***K*** is the 3 × 3 intrinsic matrix, ***I*** is a 3 × 3 identity matrix, and **0** is a 3 × 1 zero vector. M=[R|t] , where ***R*** is a 3 × 3 rotation matrix, ***t*** is a 3 × 1 translation vector, and [*x,y,z*,1]*^T^* represents the world coordinates of a point. The superscripts *c* and *p* denote parameters associated with the camera and projector, respectively. The solution of Equation 1 considering the camera and projector distortion can be expressed as Equation 2 proposed in (Vargas et al., 2023):


$$z=\frac{c_{0}+c_{1}{\text{Φ}}_{ph}\left(u,v\right)}{1+c_{2}{\text{Φ}}_{ph}\left(u,v\right)},$$

$$x=c_{3}z,$$

(2) $$y=c_{4}z,$$

where (*c*_0_ *,c*_1_ *,c*_2_ *,c*_3_ *,c*_4_) are constant coefficients that can be calibrated using the method proposed in (Vargas et al., 2023) and Φ_ph_ represents the absolute phase recovered from captured fringe images using fringe analysis methods (Zhang, 2016).

### 2.3 Mutispectral reflectance estimation

The veins of the leaf often exhibit different reflectance spectra compared to the leaf mesophyll. Therefore, we aim to estimate the spectral reflectance of the leaf and utilize it for vein segmentation. Due to the non-uniform intensity of lighting at different wavelengths, a white reference calibration method (Yu et al., 2014) is typically performed to calibrate the raw multispectral images before further processing. However, white reference calibration is limited by the fact that the estimated reflectance is influenced by the distance and surface geometry of the leaf. Consequently, in this research, we employ a multispectral reflectance estimation method that utilizes the image formation model from our previous work (Liao and Zhang, 2024).

An image formation model that estimates image intensity from the surface geometry is derived as follows (Liao and Zhang, 2024):


(3) $$\hat{J}\left(p^{\prime }\right)=\text{Ψ}\cos^{4}\alpha \left(p^{\prime }\right)f\cdot l\left(p\right)\cdot n\left(p\right).$$

Here, $p^{\prime }\in {\mathbb{R}}^{2}$ represents the projection of surface point $p\in {\mathbb{R}}^{3}$ onto the image plane, and *n*(*p*) signifies the surface normal at point *p*. $\hat{J}$ denotes the estimated image intensity. Ψ is a constant relating surface radiance to the image intensity, and *α* is the angle between the vector from the optical center of the camera to the point *p* and the optical axis of the camera. *l* is the light source model defined in the opposite direction of the light transmitted from the illumination source to *p*. *f* represents the bidirectional reflectance distribution function (BRDF). This image formation model (Equation 3) can be calibrated at each wavelength using the iterative non-linear parameter estimation technique (Liao and Zhang, 2024).

With the calibrated image formation model for each wavelength and the 3D geometry of the leaf surface, estimate the captured image intensity of the leaf at each wavelength as if the leaf has the same reflectance as the white reference can be estimated. Referencing the data provided by the manufacturer of the white reference, the estimated image intensity at each wavelength is divided by the corresponding reflectance of the white reference. This adjustment ensures that the estimated image intensity corresponds to a surface with 100% reflectance. Consequently, the reflectance of the leaf at each wavelength can be estimated using the following equation (Equation 4):


(4) $$R=\frac{J_{raw}-J_{black}}{{\hat{J}}_{100\%}},$$

where *R* is the reflectance, *J_raw_* is the original captured image, *J_black_* is the black image caused by the dark current in the camera sensors, and ${\hat{J}}_{100\%}$ is the estimated image of a 100% reflectance Lambertian surface.

### 2.4 Leaf vein segmentation

**Figure 2:**
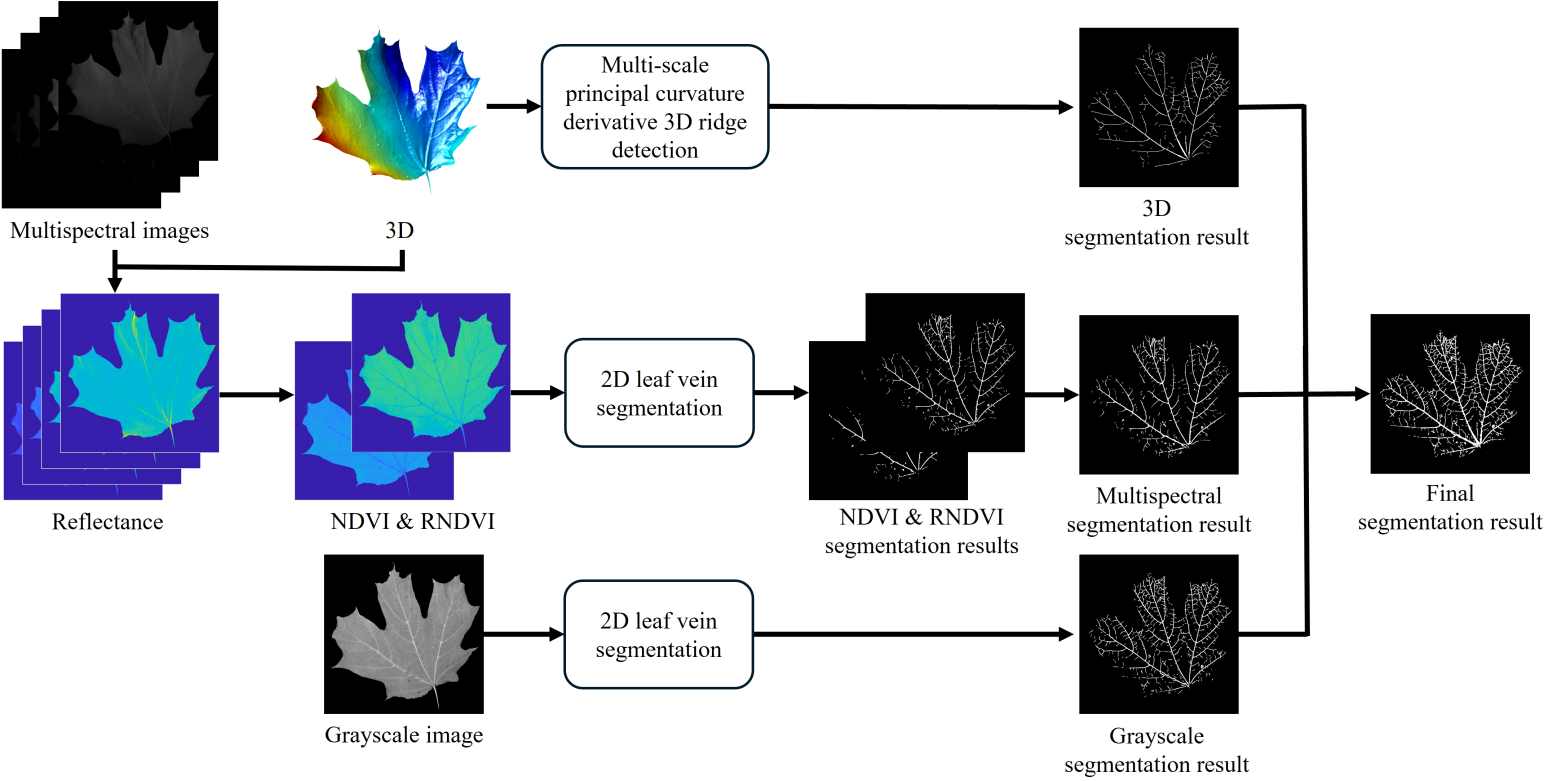
Proposed leaf vein segmentation framework.

In this subsection, the proposed leaf vein segmentation framework is introduced, as illustrated in **Figure 2**. The framework is generally divided into two main components: 2D and 3D segmentation.

For 2D segmentation, grayscale and multispectral images captured at four wavelengths: 650 nm, 700 nm, 750 nm, and 800 nm are utilized. The grayscale image is used directly for segmentation, while the multispectral images undergo preprocessing. First, reflectance at each wavelength is estimated using the method described in Section 2.3. Next, the normalized difference vegetation index (NDVI) and red-edge NDVI (RNDVI) are computed to enhance the contrast between veins and mesophyll, as these indices respond differently in vein regions (Gao et al., 2021). The indices are calculated using the following equations:


(5) $$\text{NDVI}=\frac{R_{800}-R_{650}}{R_{800}+R_{650}},$$

(6) $$\text{RNDVI}=\frac{R_{750}-R_{700}}{R_{750}+R_{700}},$$

where *R_w_* represents the reflectance at wavelength *w* nm. The computed NDVI and RNDVI values are then normalized to the range of [0,255] to standardize the data. These indices, along with the grayscale images, are used to segment leaf veins using two existing 2D methods: the Hessian matrix approach (Salima et al., 2015) and a CNN-based method (Xu et al., 2021). The segmentation results from the NDVI and RNDVI images are combined using a union operation to maximize coverage.

For the 3D segmentation, the high-resolution 3D geometry acquired via the digital fringe projection (DFP) technique is utilized. This method applies the proposed multi-scale principal curvature derivative algorithm to detect ridge-like structures on the leaf surface, capturing vein features based on their geometric properties, as described in Section 2.4.3.

The final leaf vein segmentation result is obtained by taking the union of the outputs from both 2D and 3D segmentation processes. This integration leverages the complementary strengths of 2D spectral information and 3D topographical data, aiming to enhance the robustness and accuracy of vein detection, particularly under varying environmental conditions.

#### 2.4.1 2D Hessian matrix leaf vein segmentation method

The 2D Hessian matrix leaf vein segmentation method is proposed by Salima et al. (2015). Let *J*(***x***) denote the value of a 2-dimensional data at coordinate *x* = [*x*_1_ *,x*_2_]*^T^*. The Hessian of *J*(***x***) at scale *s* is represented by a 2 × 2 matrix, defined as:


(7) $$H_{ij}\left(x,s\right)=s^{2}J\left(x\right)*\frac{\partial^{2}}{\partial x_{i}\partial x_{j}}G\left(x,s\right)\text{ for }i,j=1,2,$$

Where


(8) $$G\left(x,s\right)=\frac{1}{2\pi s^{2}}e^{-\frac{x^{T}x}{2s^{2}}}$$

is the 2-variate Gaussian function, and ∗ denotes the convolution operation. Selective enhancement of local structural features, regardless of their orientation, is achieved by examining the signs and magnitudes of the Hessian eigenvalues (Equations 7, 8). This approach relies on the shape and contrast between the brightness of the structures and their background. In cases where the data is not an image, the value’s magnitude represents the structure’s brightness.

The eigenvalues of *H*, *λ*_1_ and *λ*_2_, are obtained for each *x* through eigenvalue decomposition. The eigenvalues are sorted by their magnitudes: |*λ*_1_| ≤ |*λ*_2_|. Negative (positive) eigenvalues indicate a bright (dark) structure on a dark (bright) background. The leaf veins, which resemble tube-like structures, can be identified by the condition |*λ*_2_| ≫ |*λ*_1_|. To address variations in shape and intensity of the targeted structures, as well as image noise, the indicator functions are approximated using the vesselness function *V* (Frangi et al., 1998), which yields non-negative responses. When bright structures on a dark background,


(9) $$V=\{\begin{array}{ll} 0 & \text{if }\lambda_{2}>0\\ \text{exp }\left(-\frac{R_{B}^{2}}{2\beta^{2}}\right)\left(1-\text{exp }\left(-\frac{S^{2}}{2c^{2}}\right)\right) \end{array},$$

where $R_{B}=\lambda_{1}/\lambda_{2}$ , and $S=\sqrt{\lambda_{1}^{2}+\lambda_{2}^{2}}$ . When dark structures on a bright background, V=0 if $\lambda_{2}<0$ . The parameters β and c are thresholds that control the sensitivity to $R_{B}$ and S , respectively. A multi-scale filter response is subsequently obtained by maximizing the given enhancement function at each point across a range of scales, as follows:


(10) $$F\left(x\right)=\underset{s_{\min}\leq s\leq s_{\max}}{\max}V\left[\text{eig }H\left(x,s\right)\right],$$

where “eig” denotes the eigenvalue decomposition. The parameters *s*_max_ and *s*_min_ represent the maximum and minimum scales at which relevant structures are expected to be found. Finally, a threshold is applied to the multi-scale filter response to create a binary mask for leaf veins.

#### 2.4.2 2D CNN leaf vein segmentation method

Xu et al. (2021) presented a deep learning-based approach designed to accurately segment and analyze leaf venation networks using convolutional neural networks (CNNs). The technique leverages the U-Net architecture, which is widely utilized in image segmentation tasks, to extract detailed vein structures from high-resolution images.

The dataset consists of high-resolution scans of chemically cleared and stained leaf samples captured using a compound microscope. To enhance the visibility of fine vein structures, contrast-limited adaptive histogram equalization (CLAHE) is applied. This preprocessing step is particularly effective for leaves with low inherent contrast, as it improves the differentiation between veins and background tissue.

In this research, the trained model in the original research is utilized. For training the CNN, a set of ground-truth (GT) images was created by manually tracing vein networks within specific regions of interest (ROIs) in each leaf image. To improve model robustness, data augmentation techniques such as rotation, translation, scaling, and reflection were applied to the training dataset. The model was trained on ground-truth regions derived from over 700 leaf samples representing 50 Southeast Asian plant families, using a total of 38 CNNs trained on different subsets of these data.

To enhance segmentation accuracy and robustness, an ensemble approach was employed. Specifically, six independently trained CNN models were combined to produce the final segmentation output. Each model generated a probability map, and the ensemble averaged these maps to reduce noise and improve consistency. The resulting probability map was then thresholded to create a binary mask representing the full-width vein network.

#### 2.4.3 3D multi-scale principal curvature derivative ridge detection

Leaf veins exhibit ridge or valley-like structures. In this section, we propose a multi-scale 3D vein segmentation algorithm using derivatives of the principal curvatures. This method is inspired by the line drawings of 3D meshes (Judd et al., 2007), where lines are drawn when the surface normal changes at a locally maximal rate. In differential geometry, given a smooth surface, the shape operator, also known as the Weingarten map, is defined as:


(11) $$S\left(r\right)=-D_{r}n,$$

where *D_r_* is the directional derivative along vector ***r*** in the tangent plane, and ***n*** is the outward-facing unit normal at a point on the surface. The shape operator *S* at a point on a surface is a linear map that describes how the normal vector to the surface changes as we move along the surface. The shape operator can be represented in matrix form if we have a basis for the tangent plane of the surface at the point of interest.

At every point on the surface, the minimum and maximum curvatures, called the principal curvatures and denoted as *k*_1_ and *k*_2_, are the eigenvalues of the shape operator *S*, with |*k*_1_| ≤ |*k*_2_|. The corresponding eigenvectors *e*_1_ and *e*_2_ indicate the directions of the minimum and maximum curvatures, the principal directions, respectively. These curvatures quantify the amount the surface bends in the principal directions at that specific point. Ridges and valleys are the sets of points where the principal curvature reaches an extremum along the principal direction. The extremum occurs when $D_{e_{2}}k_{2}=0$ , where ridges occur when *k*_2_ *>* 0 and valleys occur when *k*_2<_ 0. By taking the second-order derivative, ridges and valleys are ensured when the derivatives are negative and positive, respectively. Based on the above theory, the ridges caused by the veins of the leaf are located through several steps.

Step 1: Calculate principal curvatures and directions

Compute the minimum and maximum curvature *k*_1_ and *k*_2_, and their directions *e*_1_ and *e*_2_ using the shape operator obtained from the Weingarten equations through eigenvalue decomposition.

Step 2: Estimate maximum curvature derivative

**Figure 3:**
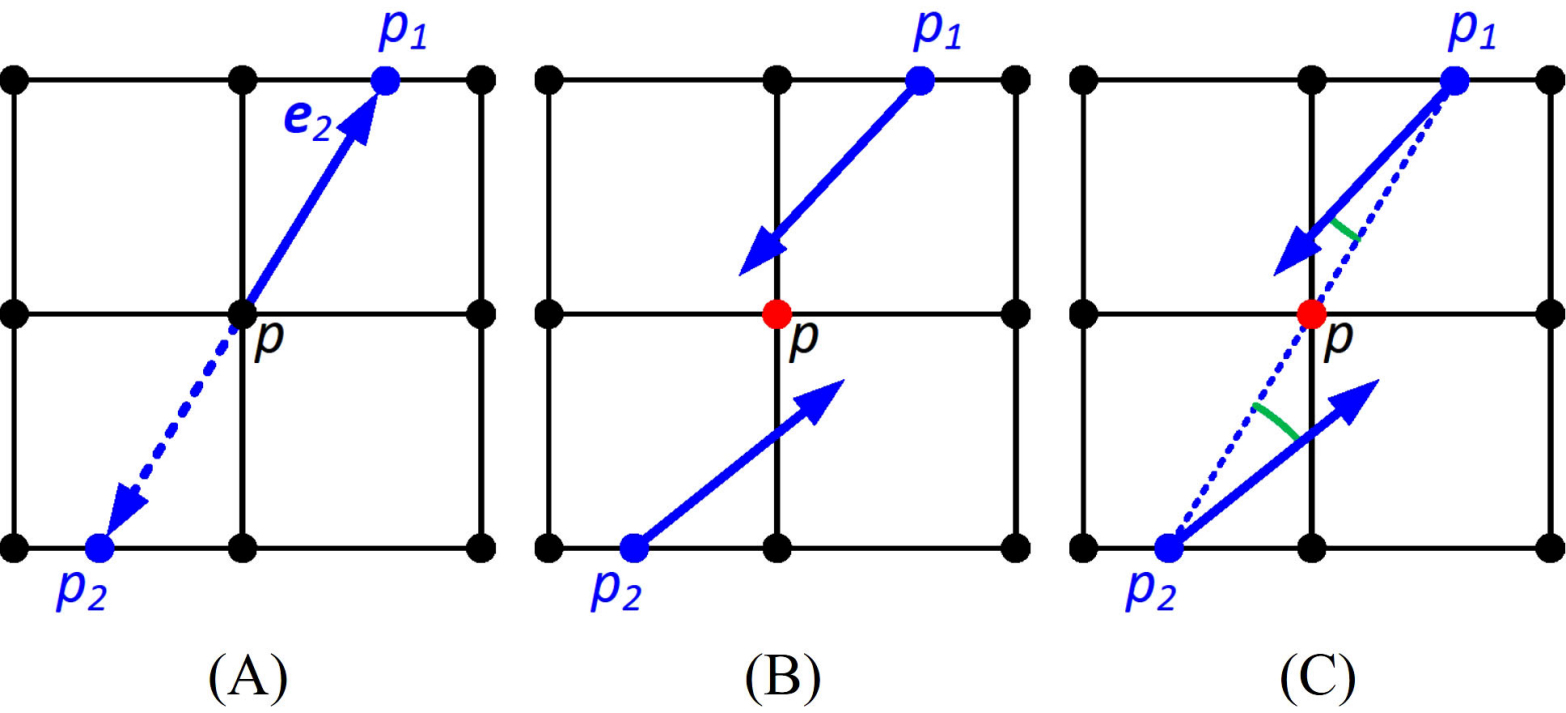
Detecting the maxima of the second-order derivative of the principal curvature. The black dots are the points of the organized point cloud. **(A)** Estimating the derivative of the maximum curvature at point *p* from *p*_1_ and *p*_2_ along the direction *e*_2_; **(B)** identifying the zero crossings of the curvature derivative by checking if the angle subtends by the maximum curvature direction of *p*_1_ and *p*_2_ is larger than 90 degrees; **(C)** identifying maxima at the extremum by checking if angles subtended by *p*_1_ and *p*_2_, and the vectors pointing from *p*_1_ and *p*_2_ to *p* are less than 90 degrees.

Estimate the maximum curvature derivative $D_{e_{2}}k_{2}$ using finite differences. Since the DFP technique generates an organized point cloud, to compute the curvature derivative at point *p*, we calculate the maximum curvature at two virtual points *p*_1_ and *p*_2_ located in the rows or columns adjacent to *p* in the direction *e*_2_. The maximum curvature at *p*_1_ and *p*_2_ is obtained by linear interpolation between their two nearest points. The differences in the maximum curvature between *p* and the two points *p*_1_ and *p*_2_ (**Figure 3A**) are then averaged.

Step 3: Flip maximum curvature directions

To maintain consistency, if we are detecting ridges, we flip the maximum curvature directions *e*_2_ so that they point in the positive derivative direction, where the maximum curvature is increasing. Conversely, we flip the maximum curvature direction in the negative derivative direction if we want to detect valleys.

Step 4: Find zero crossings of curvature derivative

Identify zero crossings of the curvature derivative where the curvature derivative is an extremum. To determine if point *p* is at a zero crossing, we check the maximum curvature direction of *p*_1_ and *p*_2_. If the angle subtended by the two maximum curvature directions is larger than 90 degrees, indicating they are pointing in different directions, then *p* is at a zero crossing, and vice versa (**Figure 3B**).

Step 5: Identify maxima at zero crossings

Zero crossings include both local minima and maxima of the second-order derivative of the maximum curvature. However, we only want the points at the maximum. Therefore, if both the curvature directions of *p*_1_ and *p*_2_ subtend angles less than 90 degrees with the vectors pointing from *p*_1_ and *p*_2_ to *p*, then the zero crossing is a maximum. Otherwise, the zero crossings are eliminated (**Figure 3C**).

Step 6: Threshold zero crossings

Since leaf veins have higher maximum curvature than other areas of the leaf, and the veins have a tube-like structure, after eliminating the minima at zero crossings, we threshold the zero crossings so that only those with sufficiently high maximum curvature and tube-like structure remain. Equation 9 is utilized by substituting *λ*_1_ and *λ*_2_ with *k*_1_ and *k*_2_, and threshold the zero crossings using the output of the vesselness function to segment the leaf veins.

The above steps can locate the loci of points at which the principal curvatures assume local maxima, in other words, the ridgelines of the ridges. However, they cannot reflect the width variation of the leaf veins. Therefore, we incorporate ridge detection with the Gaussian pyramid. A Gaussian pyramid is a multi-scale representation of a signal, created by repeatedly smoothing the signal with a Gaussian filter and then downsampling it. To generate a Gaussian pyramid in our case, start with the original point cloud, apply a Gaussian blur, downsample to reduce the resolution by a factor of two along each coordinate direction, and repeat this process for each level of the pyramid. For each level of the Gaussian pyramid, the ridge detection is performed. The higher the level in the pyramid where the vein is detected, the larger the width of the vein. The vein segmentation results are then upsampled to the original resolution using Gaussian smoothing followed by re-thresholding to mitigate jagged edges. The final leaf vein segmentation result is the union of all the vein segmentation results from each level of the pyramid.

### 2.5 Leaf vein density calculation using 3D geometry

Using the leaf vein segmentation results, the VLA can be computed, defined as the vein length per unit leaf area. Both vein length and leaf area are directly calculated from the 3D geometry obtained through our imaging system. As the grayscale image captured by our multispectral and 3D imaging system is inherently aligned with the corresponding 3D point cloud, each pixel in the grayscale image corresponds directly to a point in 3D space.

To calculate the vein length, the segmented vein structures are first skeletonized to extract the central vein lines. A breadth-first search (BFS) algorithm is employed to traverse these skeletonized vein points. The segment lengths between each point and its neighbors are computed using the 3D Euclidean distance, ensuring that the curvature of the leaf surface is accurately accounted for. The vein length is obtained by summing the lengths of all segments after completing the traversal.

For leaf area calculation, a binary leaf mask is generated through intensity thresholding applied to the grayscale and spectral images. The leaf surface area is then estimated using the 3D points corresponding to the mask. The surface is approximated by dividing it into small triangular patches. The area of each triangle is computed using the 3D coordinates of its vertices. The total leaf area is derived by summing the areas of all triangles covering the leaf surface.

Once both the vein length and leaf area are computed, the VLA is determined using:


(12) $$\text{VLA}=\frac{\text{Vein Length}}{\text{Leaf Area}}.$$

This method allows for precise VLA measurement directly from the 3D geometry, eliminating the need for leaf flattening or 2D flatbed scanning, which can introduce artifacts or inaccuracies.

## 3 Results

### 3.1 Experimental setup and parameter configuration

**Figure 4:**
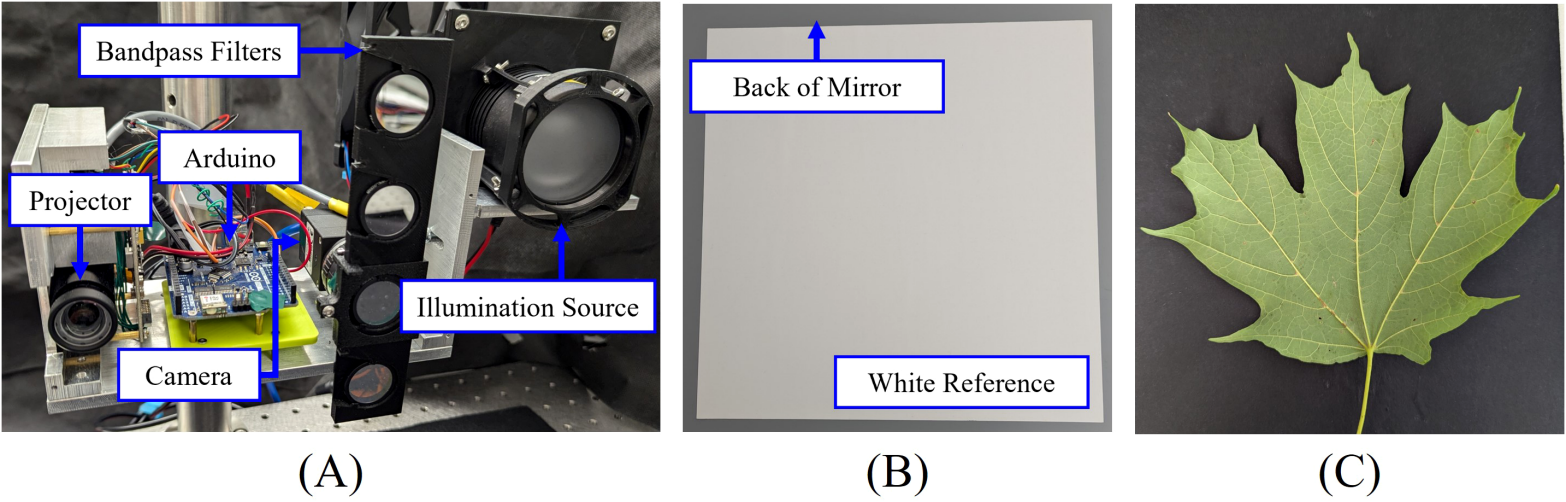
Experimental setup: **(A)** Multispectral and 3D imaging system; **(B)** white reference used for calibration; **(C)** lower side of a sugar maple leaf.

To validate the proposed methods, a multispectral and 3D imaging system is designed, as shown in **Figure 4A**. The illumination source consists of a halogen light bulb (OSRAM 64623 HLX), a plano-convex lens with a 60 mm focal length (THORLABS LA1401), and a diffuser (THORLABS DG20-600). The imaging system includes a camera (FLIR BFS-U3-28S5M-C) with a 12 mm focal length lens (Computar M1214-MP2) and a digital projector (Texas Instruments DLP 3010). The camera captures images at a resolution of 1936 × 1464 pixels with a frame rate of 120 Hz, while the projector has a resolution of 720 × 1280 pixels.

To capture multispectral images, we designed a fast-change mount for band-pass filters (THORLABS FBH650-10, FBH700-10, FBH750-10, FBH800-10) that allows for rapid switching. The camera exposure time was set to 10 ms for capturing images at wavelengths of 650 nm, 700 nm, 750 nm, and 800 nm. A PTFE diffuse reflector sheet (THORLABS PMR10P1) was used as the white reference (**Figure 4B**) for calibrating the multispectral imaging system, with the reflectance values provided by the manufacturer (THORLABS, accessed on 22 August 2024). Grayscale images were captured using the projector as the illumination source.

For the 2D Hessian matrix method, we set the scales to 1, 1.5, and 2. The parameters *β* and *c* were set to 1 and half of the maximum *λ*_2_ at scale 1, respectively, for segmenting NDVI, RNDVI, and grayscale images. The filter response threshold *F* was set to 0.5. For the 2D CNN method, we selected 6 of the 38 independently trained models with the best performance. CLAHE was applied with tile sizes of 20 and clip limits of 0.005 for grayscale images and 0.05 for NDVI and RNDVI images. The segmentation threshold was set to 20. Images were upscaled by 500% to ensure that the minimum vein width was at least 5 pixels, as recommended in the user manual.

For 3D reconstruction using the Digital Fringe Projection (DFP) technique, we employed a 3-step phaseshifted fringe pattern with multi-wavelength fringe unwrapping, utilizing a total of 6 fringe patterns to achieve high-speed 3D reconstruction. The system calibration followed the pixel-level calibration method by Vargas et al. (2023). For 3D vein segmentation, the Gaussian pyramid had 3 levels, with *β* set to 1 and *c* set to 0.4, 0.6, and 0.6 times the maximum *λ*_2_ of level 1 for levels 1 through 3, respectively. The vesselness filter threshold was set to 0.5.

All parameters for 2D and 3D segmentation were fine-tuned using a reference healthy leaf that represents typical characteristics of the dataset. This reference leaf was not included in subsequent experiments. For noise reduction, a morphological closing operation and small-area connected component removal were applied to all vein segmentation results.

For leaf vein segmentation experiments, a total of 32 leaves of varying sizes and shapes were collected from sugar maple trees (*Acer saccharum* ‘Barrett Cole’) near the FLEX Lab at Purdue University in August 2024. These leaves were immediately transferred to the lab for measurements. All imaging was conducted near windows with ambient sunlight, while a sunshade was used to block direct sunlight from the leaves. **Figures 4C** shows the lower side of an example sugar maple leaf captured using a cellphone camera.

### 3.2 Multispectral reflectance estimation

**Figure 5:**
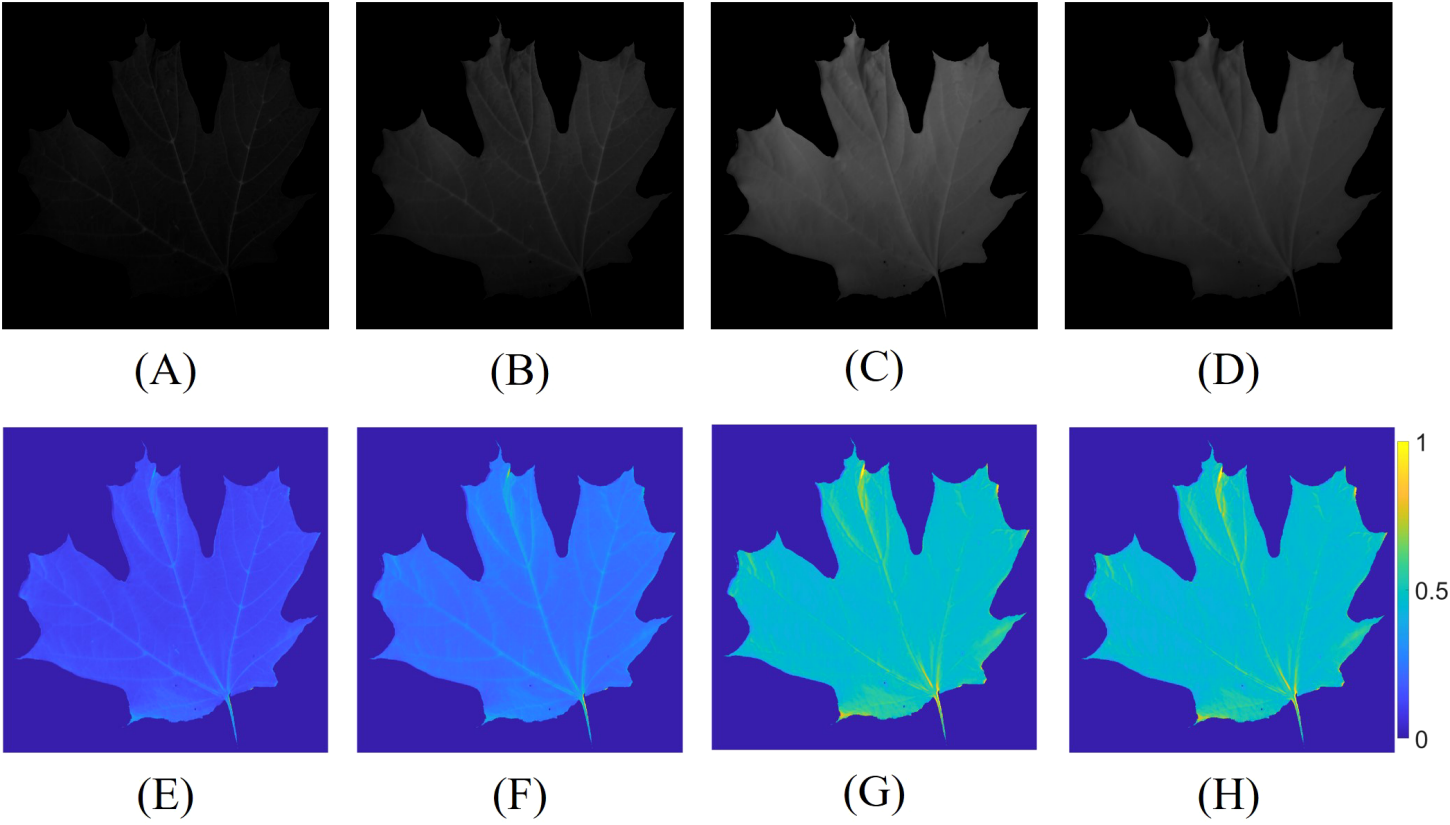
Multispectral images captured with identical camera exposure times, along with their corresponding estimated reflectance. Constant and lower exposure times were employed to reduce the measurement duration. The captured spectral images at **(A)** 650 nm; **(B)** 700 nm; **(C)** 750 nm; **(D)** 800 nm, and the estimated reflectance at **(E)** 650 nm; **(F)** 700 nm; **(G)** 750 nm; **(H)** 800 nm.

The multispectral images were first used to estimate the reflectance of the leaf, following the method outlined in Sec. 2.3. **Figures 5A–D** presents the captured multispectral images at 650 nm, 700 nm, 750 nm, and 800 nm, along with their corresponding estimated reflectance in **Figures 5E–H**. Subsequently, the reflectance values were used to calculate the NDVI and RNDVI indices using Equations 5, 6. The spectral images at 650 nm and 700 nm appear relatively dark due to the consistent camera exposure time applied across all spectral bands, which was necessary to avoid the time-consuming process of adjusting exposure settings for each frame, thereby preserving the potential for high-speed measurements. To further minimize the measurement duration, a shorter exposure time was chosen. Additionally, the inherent properties of the leaves, which exhibit lower reflectance at certain wavelengths, coupled with the relatively low intensity of the illumination source and the non-uniform camera response curve and lens transmittance at each wavelength, contributed to the darker appearance of the images at 650 nm and 700 nm.

### 3.3 Multi-scale principal curvature derivative 3D ridge detection

**Figure 6:**
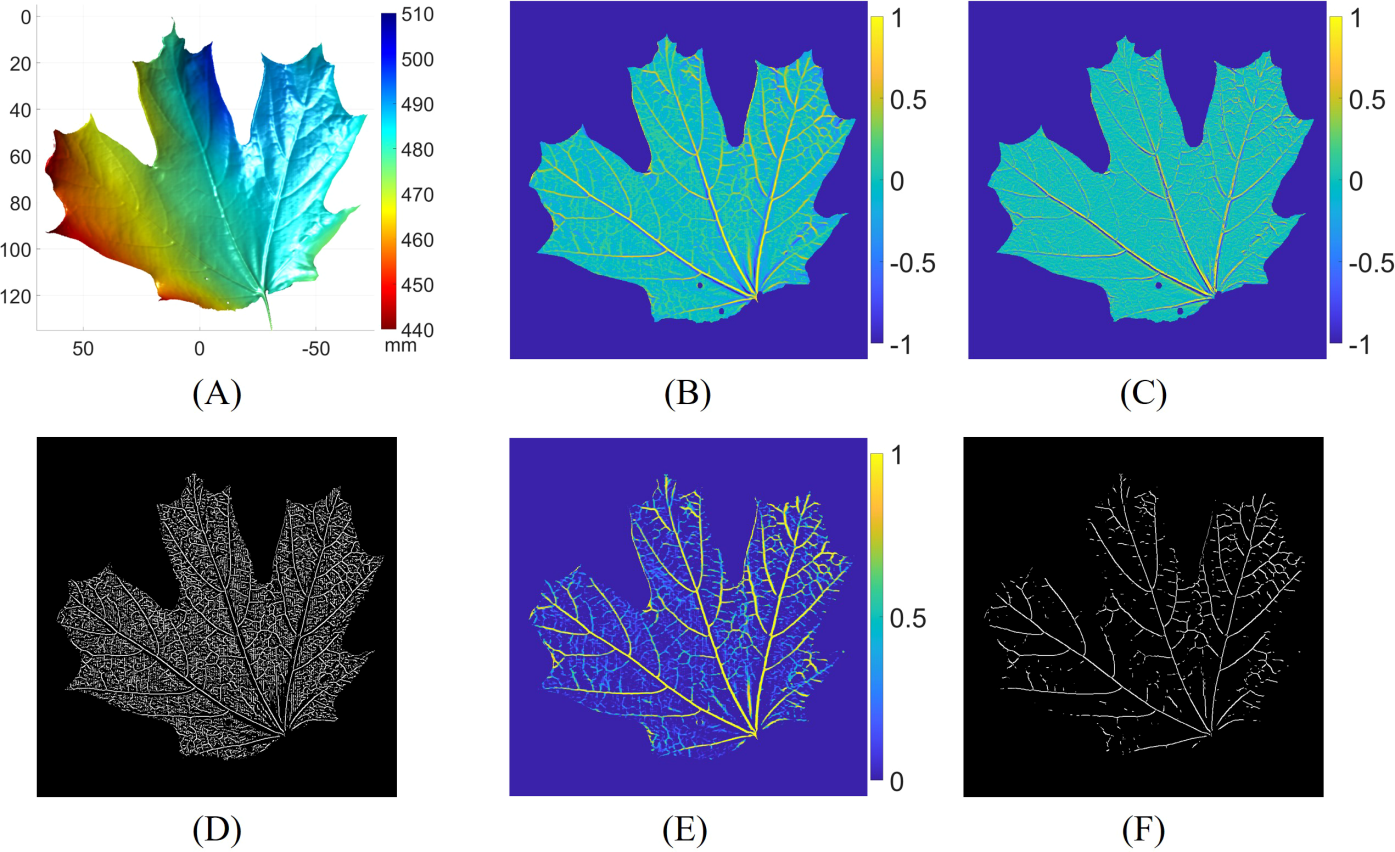
Leaf vein segmentation process at the first level of the Gaussian pyramid. **(A)** 3D surface geometry of the leaf; **(B)** maximum curvature; **(C)** maximum curvature derivative; **(D)** zero-crossings of the maximum curvature derivative; **(E)** response of the vesselness function; **(F)** leaf vein segmentation result by thresholding **(D)** with **(E)**.

This subsection shows the process of 3D leaf vein detection. **Figure 6** illustrates the ridge detection process at the first level of the Gaussian pyramid. The 3D geometry of the leaf is depicted in **Figure 6A**. The surface geometry variation is approximately 50 mm, indicating that the leaf was not flattened. The maximum curvature, calculated using the shape operator, is shown in **Figure 6B**. As expected, the vein regions of the leaf exhibit higher curvature compared to other areas. The derivative of the maximum curvature is estimated in **Figure 6C**. Across the leaf veins, the curvature derivatives display positive and negative values, indicating ridge or valley structures. The zero-crossings, where the second-order derivative of the maximum curvature achieves a local maximum, are located and displayed in **Figure 6D**. To differentiate the leaf veins from other areas, we thresholded the zero-crossings using the output of the vesselness function, based on the magnitude of the maximum and minimum curvatures, as shown in **Figure 6E**. The vesselness highlights the veins with tube-like structures. Finally, **Figure 6F** presents the 3D leaf vein segmentation result from the first level of the Gaussian pyramid.

**Figure 7:**
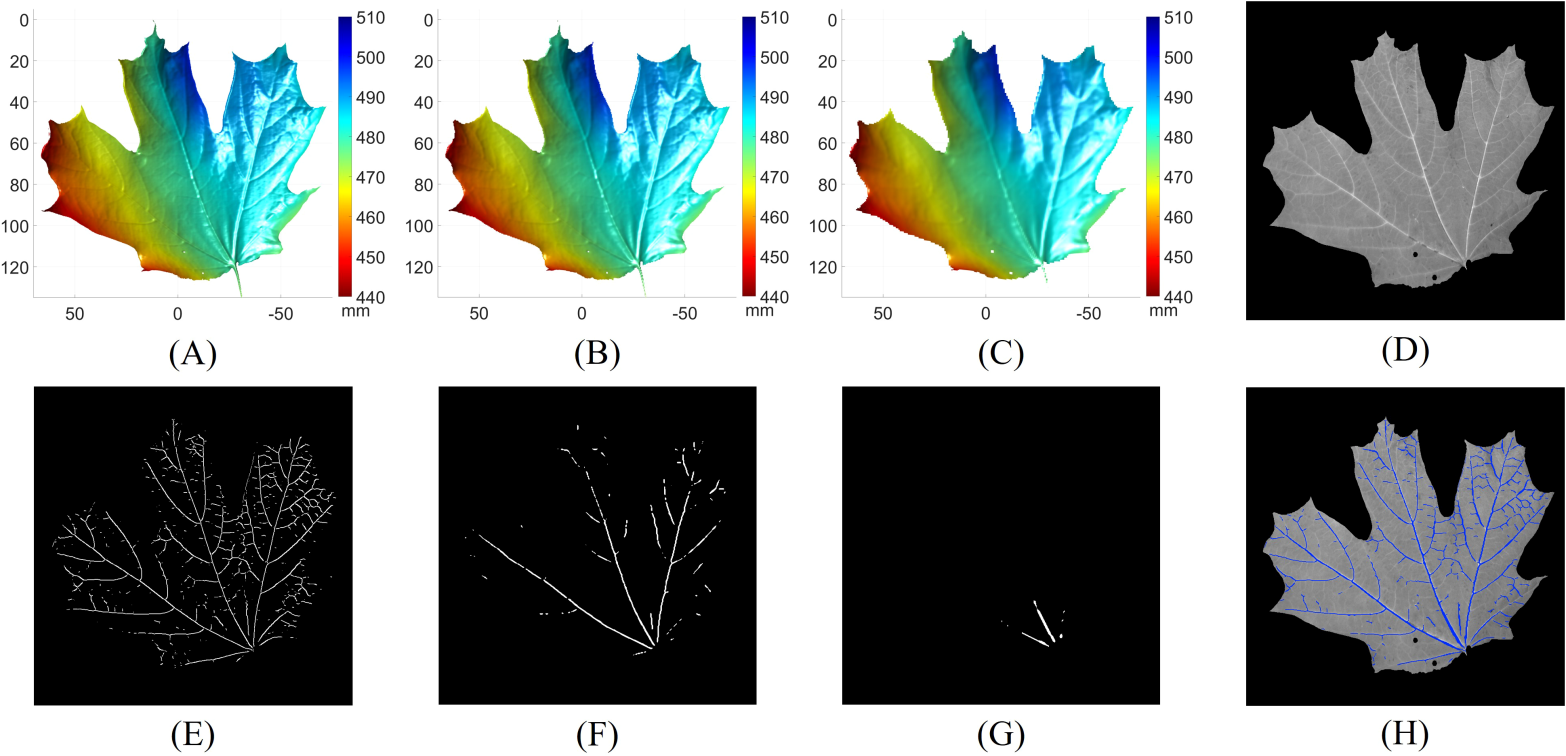
Leaf vein segmentation results using the multi-scale principal curvature derivative 3D ridge detection. **(A)** 3D geometry from the first level of the Gaussian pyramid; **(B)** 3D geometry from the second level of the Gaussian pyramid; **(C)** 3D geometry from the third level of the Gaussian pyramid; **(D)** grayscale image of the leaf; **(E)** vein segmentation result derived from **(A)**; **(F)** vein segmentation result derived from **(B)**; **(G)** vein segmentation result derived from **(C)**; **(H)** final 3D vein segmentation result (blue pixels) obtained by merging **(E–G)** overlaid on **(D)**.

**Figure 7** presents the results of leaf vein segmentation across all three levels of the 3D Gaussian pyramid. Compared to **Figures 7A**–**C** demonstrate that the leaf geometries become increasingly smoothed and downsampled, which makes smaller veins less distinguishable. As a result, the segmentation outputs shown in **Figures 7F**, **G** predominantly detect the larger veins. **Figure 7D** shows the grayscale image of the leaf. The final vein segmentation result is obtained by taking the union of the results from **Figuress 7E**–**G**, as illustrated in **Figure 7H**.

### 3.4 Combining Hessian 2D leaf vein segmentation with 3D leaf vein segmentation

**Figure 8:**
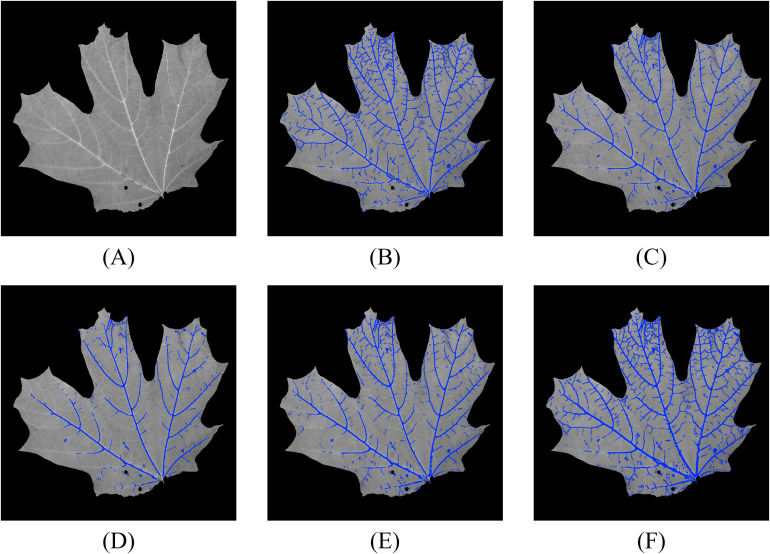
Vein segmentation results of the lower side of a sugar maple leaf using the Hessian matrix method. The segmented veins (blue pixels) are overlaid on the grayscale image. **(A)** grayscale image; **(B)** grayscale segmentation result; **(C)** NDVI segmentation result; **(D)** RNDVI segmentation result; **(E)** combined multispectral segmentation by merging **(C, D)**; **(F)** final 2D segmentation combining **(B, E)**.

We first perform leaf vein segmentation on grayscale, NDVI, and RNDVI images using the 2D Hessian matrix method. The segmentation results for one of the 32 leaves are shown in **Figure 8**. For reference, **Figure 8A** displays the grayscale image of the leaf, while **Figures 8B–F** show the corresponding vein segmentation results overlaid on **Figure 8A**. Specifically, **Figure 8B** presents the segmentation result using the grayscale image, while **Figures 8C, D** show results using NDVI and RNDVI images, respectively. **Figure 8E** illustrates the combined multispectral segmentation by merging **Figures 8C, D**. Finally, **Figure 8F** presents the comprehensive 2D segmentation result by integrating the grayscale and multispectral segmentations.

From the segmentation results, we observe that the grayscale image captures the majority of the leaf veins, except in regions with lower contrast. NDVI segmentation generally outperforms RNDVI, likely due to differences in focus levels across wavelengths. The combined multispectral segmentation demonstrates a slight improvement over NDVI and RNDVI segmentations individually. The most comprehensive results are obtained by merging both grayscale and multispectral data.

**Figure 9:**
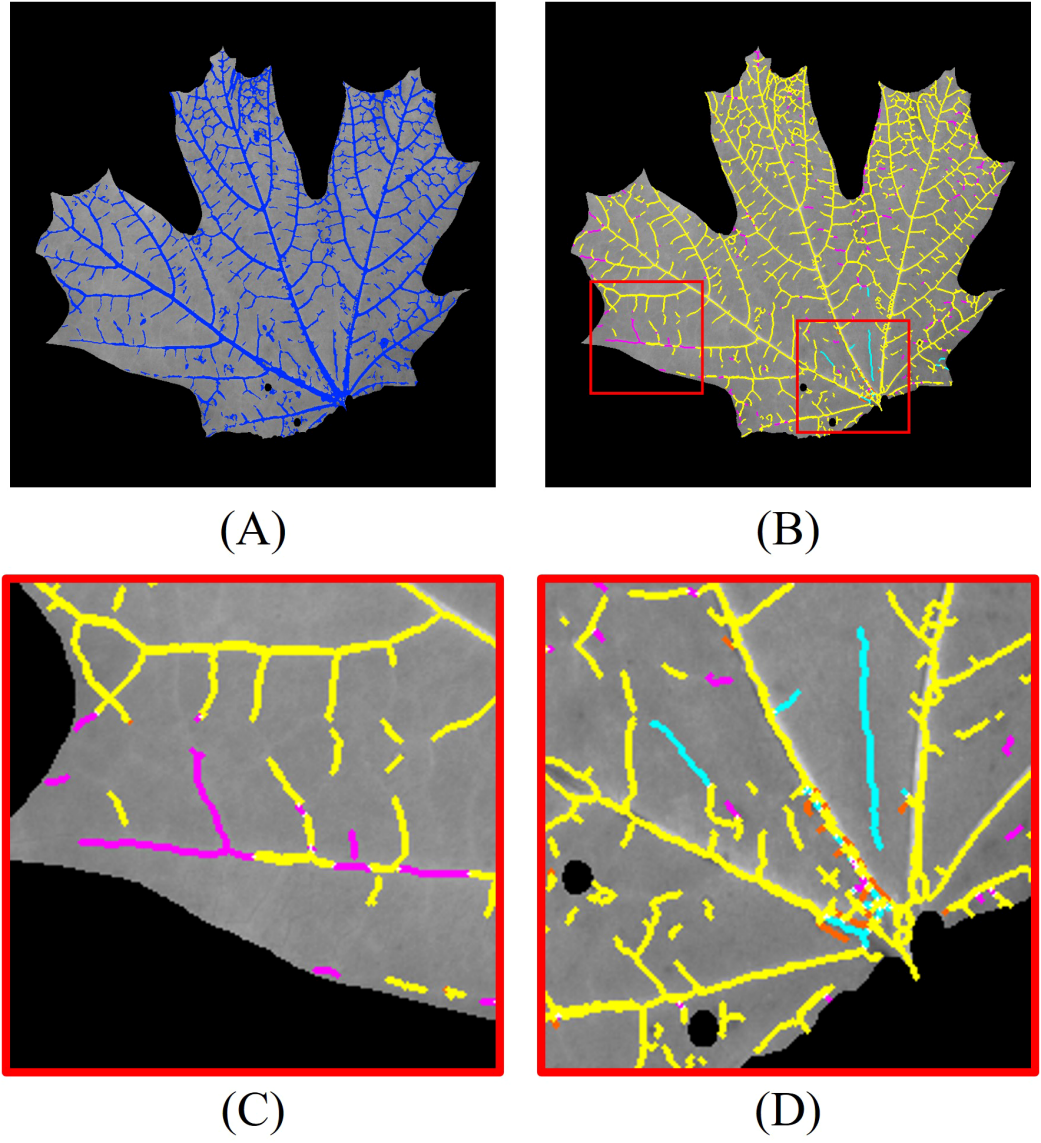
Leaf vein segmentation combining 2D Hessian and 3D segmentation. The segmented veins (blue pixels) are overlaid on the grayscale image. Yellow pixels: overlap between 2D and 2D + 3D segmentation; magenta pixels: additional positive detections by 2D + 3D segmentation; cyan pixels: additional false detections by 2D + 3D segmentation; orange pixels: veins detected only by 2D segmentation. The red rectangles are the enlarged regions. **(A)** combined 2D + 3D segmentation; **(B)** skeletonized 2D + 3D segmentation; **(C)** enlarged region highlighting improved detections using 3D segmentation; **(D)** enlarged region illustrating false positives from 3D segmentation.

To further enhance segmentation performance, we integrate 3D vein segmentation with the 2D results. The combined 2D and 3D segmentation outcomes are shown in **Figure 9A**. For detailed analysis, we skeletonize the combined segmentation result and label different segments with color codes (**Figure 9B**): yellow pixels indicate overlap between 2D and 2D + 3D segmentation, magenta pixels correspond to additional positive detections by 2D + 3D segmentation compared to 2D segmentation, cyan pixels correspond to additional false detections by 2D + 3D segmentation compared to 2D segmentation, and orange pixels correspond to veins detected only by the 2D method. For clarity, regions with pixel deviations less than two pixels after skeletonization are considered overlapping.

The 3D segmentation method provides additional vein detection in areas where 2D segmentation fails due to low contrast, as highlighted in the enlarged region in **Figure 9C**. However, it also introduces some false positives, particularly in areas with abrupt geometric variations on the leaf surface. An example of these false detections is shown in **Figure 9D**. This suggests that regions with high geometric variability or sharp deformations are more prone to producing false positives in the 3D segmentation.

**Figure 10:**
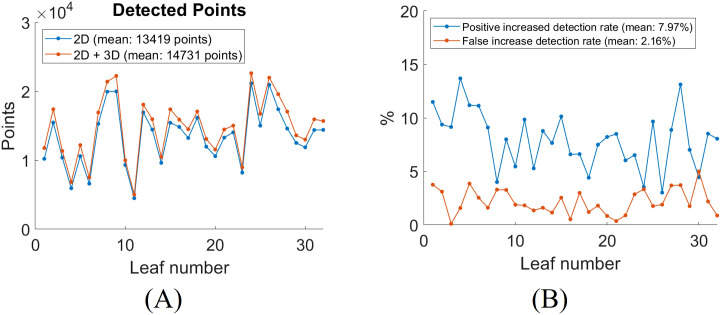
Quantitative analysis of leaf vein segmentation by incorporating 3D with 2D Hessian segmentation. **(A)** total number of detected skeletonized vein points in 2D versus 2D + 3D segmentation; **(B)** increase in detection rates after adding 3D segmentation for each leaf.

To quantitatively evaluate the impact of incorporating 3D segmentation, we compare the total number of detected vein points between the 2D and combined 2D + 3D approaches. The detected vein points are the number of pixels of the skeletonized segmentation result. The addition of 3D segmentation increases the total number of detected points, as shown in **Figure 10A**. **Figure 10B** shows the increased positive and false detection rates after integrating 3D segmentation. On average, the inclusion of 3D segmentation results in a 7.97% increase in positive detected vein points, a 2.16% increase in false detected vein points. The segmentation results for all leaves with 2D Hessian matrix method are compiled in Video 1.

### 3.5 Combining CNN 2D leaf vein segmentation with 3D leaf vein segmentation

**Figure 11:**
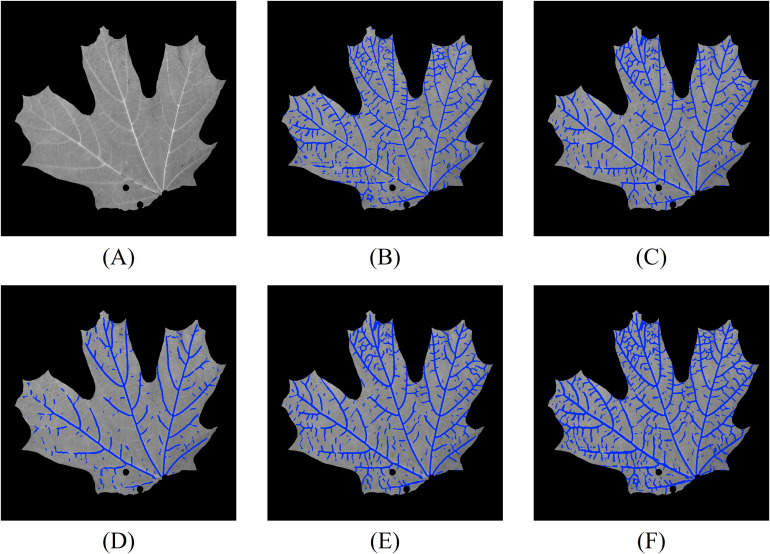
Vein segmentation results of the lower side of a sugar maple leaf using the 2D CNN method. The segmented veins (blue pixels) are overlaid on the grayscale image. **(A)** grayscale image; **(B)** grayscale segmentation result; **(C)** NDVI segmentation result; **(D)** RNDVI segmentation result; **(E)** multispectral segmentation by combining **(C, D)**; **(F)** combined 2D segmentation using **(B, E)**.

We conducted experiments using the same set of 32 leaves as in the previous analysis. **Figure 11** shows the results of leaf vein segmentation on grayscale, NDVI, and RNDVI images using the 2D CNN segmentation method. For reference, **Figure 11A** presents the grayscale image of the leaf, while **Figures 11B–F** display the vein segmentation results overlaid on **Figure 11A**. Specifically, **Figure 11B** shows the segmentation result using the grayscale image, while **Figures 11C, D** show segmentation results using NDVI and RNDVI images, respectively. **Figure 11E** represents the combined multispectral segmentation by merging **Figures 11C, D**. Finally, **Figure 11F** presents the overall 2D segmentation result by combining the grayscale and multispectral segmentation outputs.

From the segmentation results, we observe that the grayscale image captures most of the leaf veins, except in regions with lower contrast. Compared to **Figure 8E**, the multispectral segmentation shown in **Figure 11E** performs better, likely due to the CLAHE contrast enhancement step. The most comprehensive results are achieved by combining both grayscale and multispectral data. Compared to the Hessian-based method, the CNN method performs slightly better in low-contrast regions, likely because the network can extract high-level vein characteristics.

**Figure 12:**
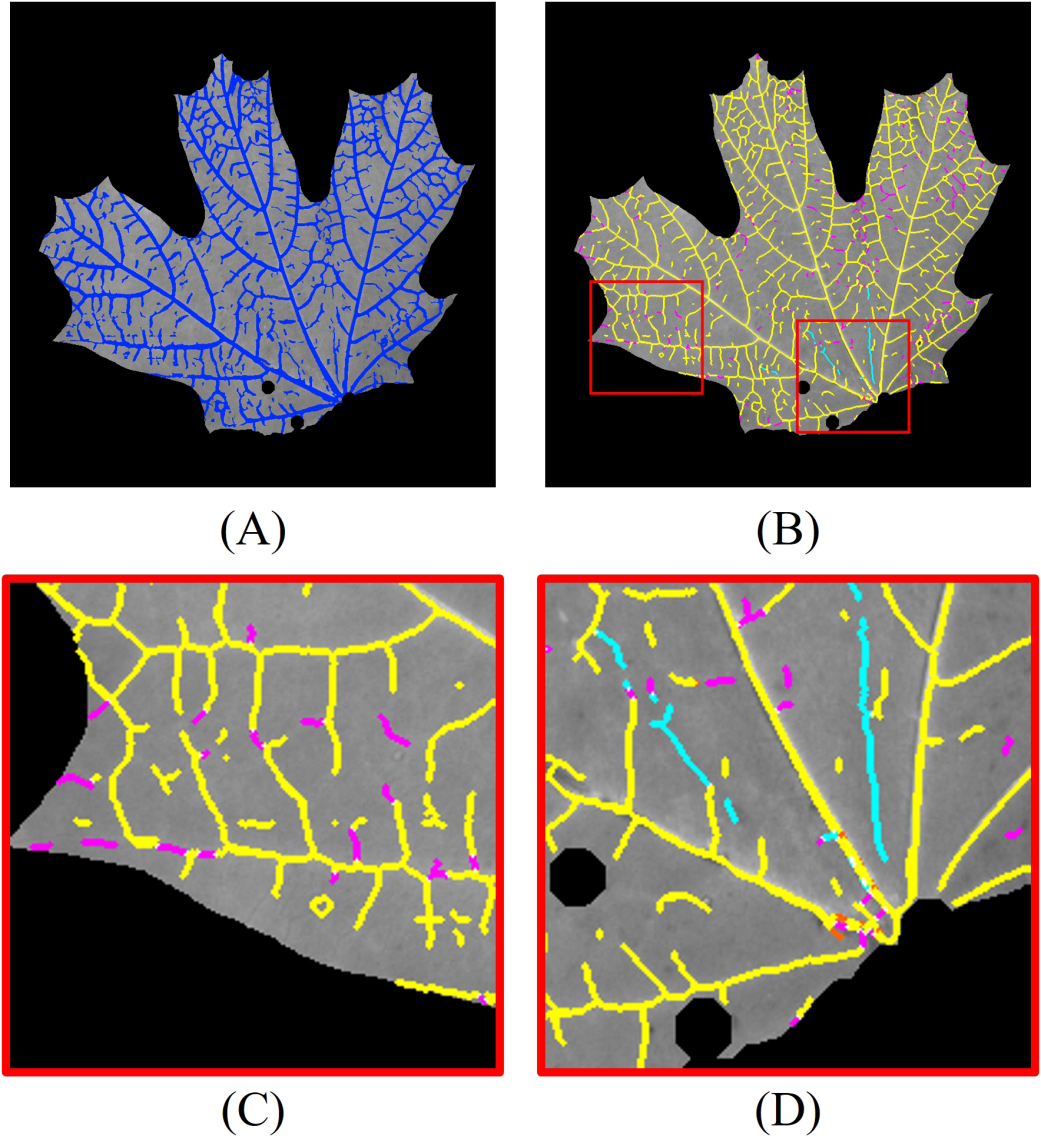
Leaf vein segmentation combining 2D CNN and 3D segmentation. The segmented veins (blue pixels) are overlaid on the grayscale image. Yellow pixels: overlap between 2D and 2D + 3D segmentation; magenta pixels: additional positive detections by 2D + 3D segmentation; cyan pixels: additional false detections by 2D + 3D segmentation; orange pixels: veins detected only by 2D segmentation. The red rectangles are the enlarged regions. **(A)** combined 2D + 3D segmentation; **(B)** skeletonized 2D + 3D segmentation; **(C)** enlarged region highlighting improved detections using 3D segmentation; **(D)** enlarged region illustrating false positives from 3D segmentation.

We also incorporated 3D vein segmentation with the 2D CNN results. The combined 2D and 3D segmentation results are shown in **Figure 12A**. For detailed analysis, we skeletonize the combined segmentation result and label different segments with color codes (**Figure 12B**): yellow pixels indicate overlap between 2D and 2D + 3D segmentation, magenta pixels correspond to additional positive detections by 2D + 3D segmentation compared to 2D segmentation, cyan pixels correspond to additional false detections by 2D + 3D segmentation compared to 2D segmentation, and orange pixels correspond to veins detected only by the 2D method. To improve visualization clarity, regions with pixel deviations of less than two pixels after skeletonization are considered overlapping.

The 3D segmentation method provides additional vein detection in areas where 2D segmentation fails due to low contrast, as highlighted in the enlarged region in **Figure 12C**. However, compared to the previous experiments, the CNN-based 2D segmentation already detects more low-contrast veins, resulting in reduced improvement from the 3D segmentation. Additionally, as in the earlier experiments, 3D segmentation introduces false detections, especially in regions with abrupt geometric changes on the leaf surface, as shown in **Figure 12D**.

**Figure 13:**
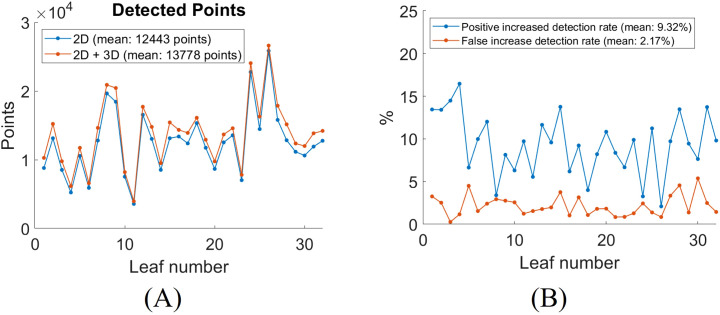
Quantitative analysis of leaf vein segmentation by incorporating 3D with 2D CNN segmentation. **(A)** total number of detected skeletonized vein points in 2D versus 2D + 3D segmentation; **(B)** increase in detection rates after adding 3D segmentation for each leaf.

To quantitatively evaluate the impact of incorporating 3D segmentation, we compared the total number of detected vein points between the 2D and combined 2D + 3D segmentation approaches. The results for all 32 leaves are summarized in **Figure 13A**. On average, the inclusion of 3D segmentation results in a 9.32% increase in positive detected vein points, and a 2.17% increase in false detected vein points (**Figure 13B**). The segmentation results for all leaves using the 2D CNN method are compiled in Video 2.

From the experiments in Sec. 3.4 and Sec. 3.5, we observe that integrating 3D data improves segmentation results but introduces additional false detections. Thus, users can assess whether the benefits of 3D integration justify its application for their specific needs. In subsequent experiments, beyond vein segmentation, we utilize the precise geometric data from 3D imaging to calculate an important plant trait: VLA.

### 3.6 Leaf vein density measurement using 3D geometry

Traditional methods for calculating VLA often involve flattening and 2D scanning the leaf using a flatbed scanner with known dpi. However, the requirement for flattening can damage the leaf, and leaves with high curvature are difficult to flatten completely, which compromises accuracy. Additionally, the flattening and scanning process can be time-consuming. By utilizing high-resolution 3D information of the leaf, we can directly measure leaf traits, such as VLA, without the need for flattening.

**Figure 14:**
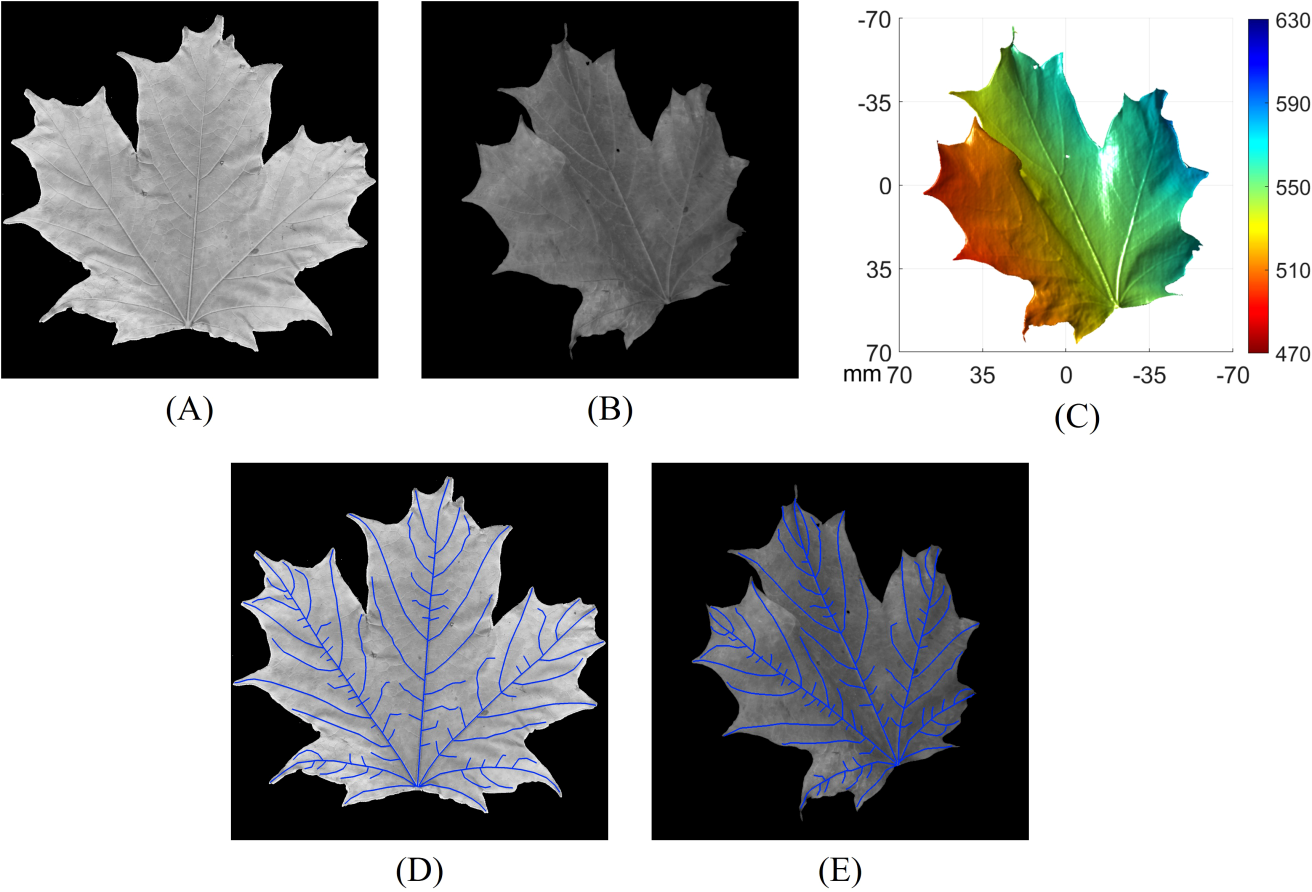
Comparison of vein tracing on the same leaf using various methods. **(A)** Flattened leaf image captured using a flatbed scanner; **(B)** non-flattened leaf image; **(C)** 3D geometry of the leaf corresponding to **(B)**; **(D)** hand-traced veins on the flattened leaf image; **(E)** hand-traced veins on the non-flattened leaf image.

To verify the accuracy of using 3D geometry for calculating VLA on non-flattened leaves with arbitrary orientation, we conducted an experiment on an additionally collected sugar maple leaf to measure VLA. First, the non-flattened leaf was imaged using our high-resolution 3D imaging system to capture both its 3D geometry and the corresponding grayscale image. Subsequently, the same leaf was flattened and scanned using a flatbed scanner (HP MFP E87660) with a resolution of 600 dpi. To eliminate the influence of segmentation performance, we carefully hand-traced the skeletons of the major veins with clear start and end points on both the flattened and non-flattened leaf images. The same veins were traced on both images to ensure consistency. **Figure 14A** displays the flattened leaf image captured using a flatbed scanner, while **Figure 14B** shows the non-flattened leaf image, with its corresponding 3D geometry illustrated in **Figure 14C**. For the vein tracing results, **Figure 14D** presents the hand-traced veins on the flattened leaf image, and **Figure 14E** shows the hand-traced veins on the non-flattened leaf image. The VLA of the leaf was calculated using three different approaches:

1. Flattened 2D method: Use the hand-traced veins on the flattened flatbed-scanned image with the scanner’s dpi for VLA calculation.
2. Non-flattened 3D method: Use the hand-traced veins on the non-flattened leaf images, with the corresponding 3D geometry, and calculate VLA as described in Section 2.5.
3. Non-flattened 2D method: Use the hand-traced veins on the non-flattened leaf images, with the camera’s dpi at the average distance of the leaf surface for VLA calculation.

The measured vein length, leaf area, and VLA from the three approaches are summarized in **Table 1**.

**Table 1:** Comparison of leaf vein length, leaf area, and VLA measurements using three different approaches.

	VL (mm)	LA (mm^2^)	VLA (mm/mm^2^)
Flattened 2D	2013.3	11921.4	0.169
Non-flattened 3D	1957.5	11604.8	0.169
Non-flattened 2D	1725.5	9237.4	0.187

The results show that the non-flattened 3D method achieves approximately the same VLA compared to the flattened 2D method. The vein length and leaf area measured by the non-flattened 3D method are slightly lower, with differences of 2.8% and 2.7% respectively, compared to the flattened 2D method. These differences are relatively minor, considering potential variations due to hand tracing, measurement artifacts, occlusions, and imperfect leaf flattening. In contrast, the differences between the flattened 2D method and the non-flattened 2D method are significantly larger, with discrepancies of 14.3%, 22.5%, and 10.6% for vein length, leaf area, and VLA, respectively. The greater discrepancy, particularly in leaf area, underscores the accuracy of the non-flattened 3D method and highlights the limitations of using 2D imaging without flattening. These findings demonstrate that high-resolution 3D imaging can achieve accuracy comparable to 2D flattening approaches in measuring vein length, leaf area, and VLA. This provides a viable alternative for calculating leaf vein traits without the need for flattening, which is particularly beneficial in unstructured environments.

## 4 Discussion

The results of this study underscore both the strengths and limitations of integrating high-resolution 3D imaging with traditional 2D techniques for leaf vein segmentation and VLA measurement. Our initial hypothesis–that incorporating 3D topographical information would enhance vein detection, particularly under suboptimal imaging conditions–was partially supported by our findings. The 3D imaging system successfully captured detailed geometric features that 2D methods struggled to identify, leading to improved detection of veins, especially in low-contrast regions where traditional approaches often fail. By leveraging the additional depth information, our system was able to distinguish veins more effectively from the surrounding mesophyll, resulting in a notable increase in the number of detected vein points. However, while the inclusion of 3D data enhanced segmentation performance, it also introduced some challenges. Specifically, the increased sensitivity to surface irregularities, such as variations in mesophyll geometry, led to a higher rate of false positives. Therefore, it will be up to the users to determine whether the integration of 3D segmentation provides sufficient value for their specific application.

Beyond vein segmentation, the 3D imaging system demonstrated clear advantages in direct trait measurements. Utilizing high-resolution depth maps, we were able to derive key metrics such as leaf area, vein length, and VLA directly from the 3D geometry. These measurements were consistent with those obtained from traditional 2D flatbed scans, highlighting the potential of 3D imaging for non-invasive phenotyping. This is particularly beneficial in field conditions where leaves cannot be chemically cleared or flattened, allowing for rapid and non-destructive data acquisition. However, the 3D method might show limitations in cases where occlusions occurred on the leaf surface. For instance, overlapping leaves, curled edges, or dense trichomes introduced artifacts in the 3D point cloud, complicating accurate segmentation and trait extraction.

Additionally, while the proposed method was validated on sugar maple leaves, further exploration is needed to assess its generalizability to other species. Different leaf structures, such as denser venation networks or more pronounced surface undulations, may require adjustments to the imaging parameters or segmentation algorithms. Future studies should focus on refining these techniques to enhance robustness across a wider range of plant species.

In summary, integrating high-resolution 3D imaging with conventional 2D methods improved vein segmentation detection rate, particularly in challenging conditions. While it introduced some false positives, the overall benefits in vein detection and direct trait measurement suggest that 3D imaging is a promising tool for precision agriculture. The ability to measure complex leaf traits non-destructively highlights its potential for high-throughput phenotyping in natural, unstructured environments.

## 5 Conclusion

This study developed and validated a multispectral, high-resolution 3D imaging system to investigate whether integrating 3D topographical data with traditional 2D imaging methods could improve leaf vein segmentation and VLA measurement in non-flattened sugar maple leaves. The results partially supported our hypothesis: combining 3D imaging with 2D techniques led to increased positive vein detection rates of 7.97% and 9.32%, and increased false vein detection rates of 2.16% and 2.17% for the 2D Hessian matrix and CNN-based methods, respectively. Therefore, users can decide if 3D segmentation adds sufficient value for their application. Despite these challenges, the 3D imaging system demonstrated clear strengths in direct trait measurements. It provided accurate assessments of vein length, leaf area, and VLA, with less than a 3% discrepancy compared to traditional flattened 2D methods without requiring destructive sample preparation. This capability is particularly valuable in field conditions, where non-invasive and high-throughput phenotyping is essential. These findings highlight the potential of the proposed 3D imaging system as a robust tool for precision agriculture, particularly in applications where traditional methods are impractical. Future research should focus on optimizing the 3D segmentation algorithms to reduce false positives and extend the system’s applicability to a broader range of plant species with diverse leaf architectures. This will ultimately advance automated phenotyping technologies, enabling more accurate and efficient assessments of plant traits critical for sustainable agricultural practices.

## Data Availability

The raw data supporting the conclusions of this article will be made available by the authors, without undue reservation.
